# Oxidised zirconium heads show a lower linear wear rate than 28-mm metal heads against XLPE: a retrospective analysis

**DOI:** 10.1177/11207000251412753

**Published:** 2026-02-25

**Authors:** Amit Atrey, Zoe Thompson, Veronica Pentland, Richard McCalden, Kevin Gargan, Douglas Naudie, James P Waddell, Graeme Hoite, Amir Khoshbin

**Affiliations:** 1 Division of Orthopaedics, University of Toronto, Toronto, ON, Canada; 2 St Michael’s Hospital, Toronto, ON, Canada; 3 Western University, London, ON, Canada; 4 University Hospital, London Health Sciences Centre, London, ON, Canada; 5 Department of Orthopedic Surgery, Omaha, University of Nebraska Medical Center, Nebraska

**Keywords:** Hip arthroplasty, hip replacement, oxidised linear wear, OXINIUM, tribology

## Abstract

**Aims:**

The use of highly cross-linked polyethylene (XLPE) on the acetabulum has undoubtedly improved the wear rates and hence survivorship of total hip arthroplasty (THA). However, the importance of head tribology is not known. Oxidised zirconium (OxZr) was introduced to offer the lower wear rates of ceramic but the strength of metal. Whether OxZr heads have any purported benefits in terms of lowering wear is not known. This study therefore aims to compare wear rates of XLPE against metal and OxZr heads over a 10-year period.

**Methods:**

A previous study has suggested we need a minimum 95 cases to show a statistically significant difference. Thus, in this 10-year retrospective analysis we included 95 subjects with a primary THA with a 28-mm OxZr and 131 patients with a 28-mm cobalt-chrome (CoCr) head, both against XLPE liners.

Linear and volumetric wear rate comparisons were performed using a validated 2-dimensional stereoisometric analysis by an independent centre.

**Results:**

There were no significant differences for any of the patient demographics between the 2 groups, with >60% women in both groups and normally distributed age. Cup inclination angle was also comparable between the 2 groups.

While there was no difference in volumetric wear rates (*p* = 0.7377), the annual linear wear rate for OxZr was 0.030 mm (95% CI, 0.024–0.036) compared with 0.046 mm (95% CI, 0.038–0.054) for CoCr, a statistically significant difference (*p* = 0.0002)

**Conclusions:**

The widespread use of XLPE has revolutionised THA. This study is the first to show a statistically significantly lower wear rate with the use of OxZr. Whether this is enough to justify the cost differential and whether it translates to better implant survivorship is still to be determined. But if wear limiting wear rates is associated with decreasing revision rates, the use of ceramicised metal may be of clinical benefit.

## Introduction

Highly cross-linked polyethylene has dramatically changed the wear rates of hard-on-soft bearings in total hip arthroplasty (THA).^1–3^ Most reports show that it results in up to 4 times less wear than with conventional polyethylene and lower wear rates than the reported 0.1-mm/year threshold associated with osteolysis.^4–9^

Since then, much work has concentrated on the opposite side of the bearing combination – the femoral head. It has been postulated that the better scratch profile, surface smoothness and wettability of ceramic heads when compared to traditional cobalt chromium (CoCr) heads may decrease polyethylene debris/wear and therefore result in less osteolysis/aseptic loosening.^10–13^

This has led many surgeons to convert to the use of ceramic heads instead of metal despite an increased economic cost,^14^ even though the majority of the evidence indicates there are not lower wear rates with a ceramic head and only a recent meta-analysis indicates a possible trend to lower wear.^13,15–18^ It is also thought that ceramic heads will lower the incidence of mechanically activated crevice corrosion (MACC) or trunnionosis.^19^ This is often cited as a major reason for the rise in the use of ceramic heads for primary THA.^20^

The risks of using ceramic heads include their brittleness, leading to a relatively high incidence of fracture. Even though third and fourth generation ceramic heads report lower fracture rates, reports of varying fracture rates between 0.01% and 1.5% persist.^21,22^

Oxidised zirconium (OxZr) was introduced to reduce polyethylene wear (because of the ceramic outer layer) while the metal core aimed to eliminate the potential for catastrophic fracture.^23^

There have been encouraging data showing lower wear rates in the short term of OxZr against ultra-high molecular weight (UHMW) polyethylene (4 years),^24^ and an RCT comparing wear of metal, ceramic and OxZr against XLPE (highly cross-linked polyethylene) at 5 and 7 years.^25,26^ Both of these studies have been underpowered and have follow-up only in the early phase of survivorship. Of 2 10-year RCT RSA analyses, 1 found no statistical difference between metal and OXINIUM (Smith+Nephew, Memphis, TN, USA) heads at 10 years,^27^ while a second demonstrated a trend towards lower wear rates in favour of OxZr, although this finding was not statistically significant.^28^

Conversely, A previously published article from this study group found there were lower wear rates with the oxidised zirconium head than with metal heads, but the study remained underpowered and the results were not statistically significant.

To discover whether there was a significant decrease in XLPE wear with OxZr compared with metal heads, this study was appropriately powered (based on our previous study).

We therefore present a minimum 10-year follow-up of 2 separate bearing combinations to assess if there is any statistical difference in the wear rates of cobalt-chrome metal heads versus OxZr heads with the same contemporary XLPE.

## Methodology

In this retrospective study, we acquired Ethical Board approval (#19-275) to perform a validated assessment on the wear rates of 2 different bearing surfaces.

Inclusion criteria consisted of patients aged 18–65 years undergoing THR for primary arthritis. Exclusion criteria included a history of hip joint sepsis, primary or secondary malignancy in the involved hip, secondary osteoarthritis due to inflammatory arthropathy, fracture of the femoral neck, and bone deficiency requiring the use of autologous or allograft bone.

Patients were recruited between 2008 and 2013 from three academic centres. These were St Michael’s Hospital (Toronto, Canada), Western University (London, Canada) and the University of Nebraska (Lincoln, Omaha, NE, USA). Patients were followed up for a minimum of 10 years.

Our previous study looked at 4 bearing types including OxZr and CoCr against conventional ultra-high-molecular-weight polyethylene (UHWMWPE) and XLPE.^29^ We found a trend to lower XLPE wear rate for OxZr heads compared with CoCr. However, this difference could not be considered statistically significant based on the low cohort numbers (23–25 patients in each arm of the study).

From this study, a power calculation was performed and we predicted at what difference a statistically significant difference may be detectable if the cohort size was increased to a minimum of 95 patients.

We therefore collated immediate postoperative and 10-year postoperative images of patients who had undergone THA with either a CoCr or OxZr head. As our initial cohorts included 28-mm heads, this analysis was limited to this head size alone. All implants were made by Smith+Nephew (Memphis, TE, USA) and were a Porous Synergy stem and hydroxyapatite (HA) coated Reflection cup.

The primary outcome assessed is average linear wear rates over 10 years and the secondary outcome is volumetric wear rates.

A standardised anteroposterior (AP) of the pelvis and a lateral view of the hip were taken immediately postoperatively and then again at 10 years, and radiographic assessment was performed to assess steady state wear. While there would be some initial creep, this was present in both groups.

### Radiological assessment

Radiological assessment of wear was performed by a blinded, independently contracted company that had developed the Polyware digital analysis program (Draftware inc, Vevay, IN, USA). Polyware is a software application that creates a 3-dimensional model of implants based on AP and lateral radiographs.^3^ The process has been validated,^13^ with an inter-observer error at 95% estimated to be ±0.007679 and has previously been reported to be more reliable than 2-dimensional analysis.^13,30^ Calibration is performed using standardised 28-mm femoral heads and the program is compatible with the Smith+Nephew Reflection acetabular component. Changes in volumetric and linear wear, as well as directional analysis, are calculated by the program. The linear wear accuracy of Polyware is ±0.015 mm and the volumetric is validated as ±0.13 mm^3^.^14^ We also assessed acetabular inclination (via the automated program as well as via manual assessment with reference to the trans-ischial line). Any osteolysis or radiolucent lines (RLLs) were assessed using the lines of Gruen et al.^31^ and of DeLee and Charnley^32^ for the femoral and acetabular components, respectively.

The secondary outcome was volumetric wear (mm/cm^3^).

### Statistical analysis

For demographics and wear results, means and standard deviations (SDs) were calculated and are listed in the results. For comparison between the 2 groups an unpaired student’s *t-*test was conducted with a 2-tailed hypothesis and a significance value of 0.05. To determine normality of samples, the Kolmogorov-Smirnov test was used. For non-normally distributed samples a two-tailed Mann-Whitney U-test with a significance value of 0.05 was conducted to determine significance.

This manuscript was prepared following the STROBE (Strengthening the Reporting of Observational Studies in Epidemiology) checklist for observational studies.

## Results

### Demographics

All patients included in this study received primary total hip arthroplasty and had no evidence of osteolysis as part of their clinical picture. 95 patients were assigned to the oxinium group and 131 to the cobalt group. Age and sex data were collected for all patients and the results are listed in Table 1. Both groups were female dominated with >60% of patients being female in both groups. When comparing age across the 2 groups there was no statistically significant difference between the means of the 2 groups. All age data were normally distributed.

**Table 1.** Demographic data comparison between groups. Standard deviation listed in brackets following mean.

	OXINIUM Group	Cobalt Group	*p*-value
Sample (*n*)	95	131	-
Age (years)	54.6 (± 9.13)	57.7 (± 12.5)	0.135
% Female patients	64.2	61.1	-
Average BMI (kg/m^2^)	26.3 (± -4.2)	25.9 (± 5.3)	0.142

BMI, body mass index.

All cups were investigated irrespective of the cup inclination angle.

All cups were HA-coated Reflection with XLPE liners and 87% were high offset Synergy stems (Smith+Nephew) with the remainder standard offset Synergy stems.

### Wear analysis

**Figure 1.**
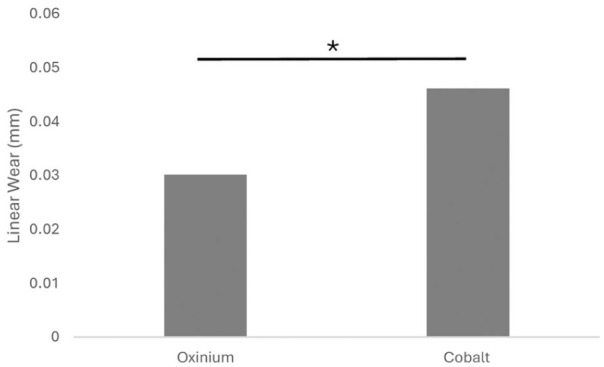
Graph of average linear wear of OxZr versus metal heads.

**Figure 2.**
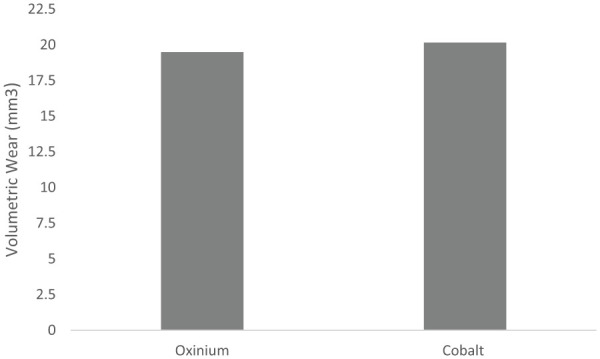
Graph of average volumetric wear of OxZr versus metal heads.

Outcomes for wear over time were calculated in terms of both linear and volumetric wear per year, and summary results are listed in Table 2. Volumetric wear values were normally distributed in both groups and statistical analysis with a *t*-test revealed no significant difference between the means of the 2 groups (Table 1) (Figures 1 and 2).

**Table 2.** Comparison of annual linear and volumetric wear between groups. Standard deviation listed in brackets following mean.

	OXINIUM Group	Cobalt Group	*p-*value
Linear wear/year	0.0301 (± 0.0276)	0.0461 (± 0.0416)	**0.0002**
Volumetric wear/year	19.48 (± 13.82)	20.15 (± 15.46)	0.7377

Note: value in bold indicates statistical significance.

Conversely, linear wear rates were non-normally distributed and thus the Mann-Whitney U-test was used to show a statistically significant difference between the means of the 2 groups with the cobalt group having higher wear rates per year when compared to the OXINIUM group (Table 2) (Figure 1).

## Discussion

This 10-year multicentre retrospective follow-up analysis has demonstrated that there were statistically significant lower wear rates of XLPE when combined with an oxidised zirconium head than with a cobalt-chrome head. This assessment was performed with a validated calibrated imaging tool performed by a blinded secondary company.

To our knowledge, this is the first 10-year follow-up to show such a statistically significant difference.

Prior to the introduction of XLPE to THA, osteolysis and subsequent implant aseptic loosening was often the leading cause of revision.^33^ XLPE has led to a dramatic change in implant survivorship by a reduction in polyethylene wear.^4,34^

After widespread uptake in the use of XLPE, the linear wear rates of polyethylene dropped below what was thought to be a threshold limit of 0.1 mm/year.^9^ Wear above this limit is thought to be the instigator of osteolysis and hence aseptic loosening. When XLPE is used, neither metal nor OxZr will come close to this threshold for wear but finding the lowest wear rate and related clinical significance is the goal of this study.

XLPE usage in hip arthroplasty has resulted in only anecdotal cases of osteolysis and the associated lower revision rates.^35^ As a result, the use of “hard on soft” is now the predominant bearing combination in North America with the “soft” being XLPE.^36^

However, there is controversy over the ideal partnering head. While there are theoretical benefits to the use of a ceramic head (over a metal head) such as better scratch profile and better wettability,^37,38^ only 1 study has demonstrated any improvement in conventional polyethylene,^39^ and this has not transferred to any meaningful *in vivo* improvement in wear profile with XLPE. Conversely, the brittle nature of ceramic means there remains a risk fracture of these heads. Even with third generation ceramics, this is reported to be 0.79/1000 cases.^30^ Oxidised zirconium was therefore developed as a proprietary material by a single vendor. This offers the benefits of a ceramic surface with the strength of metal.

**Figure 3.**
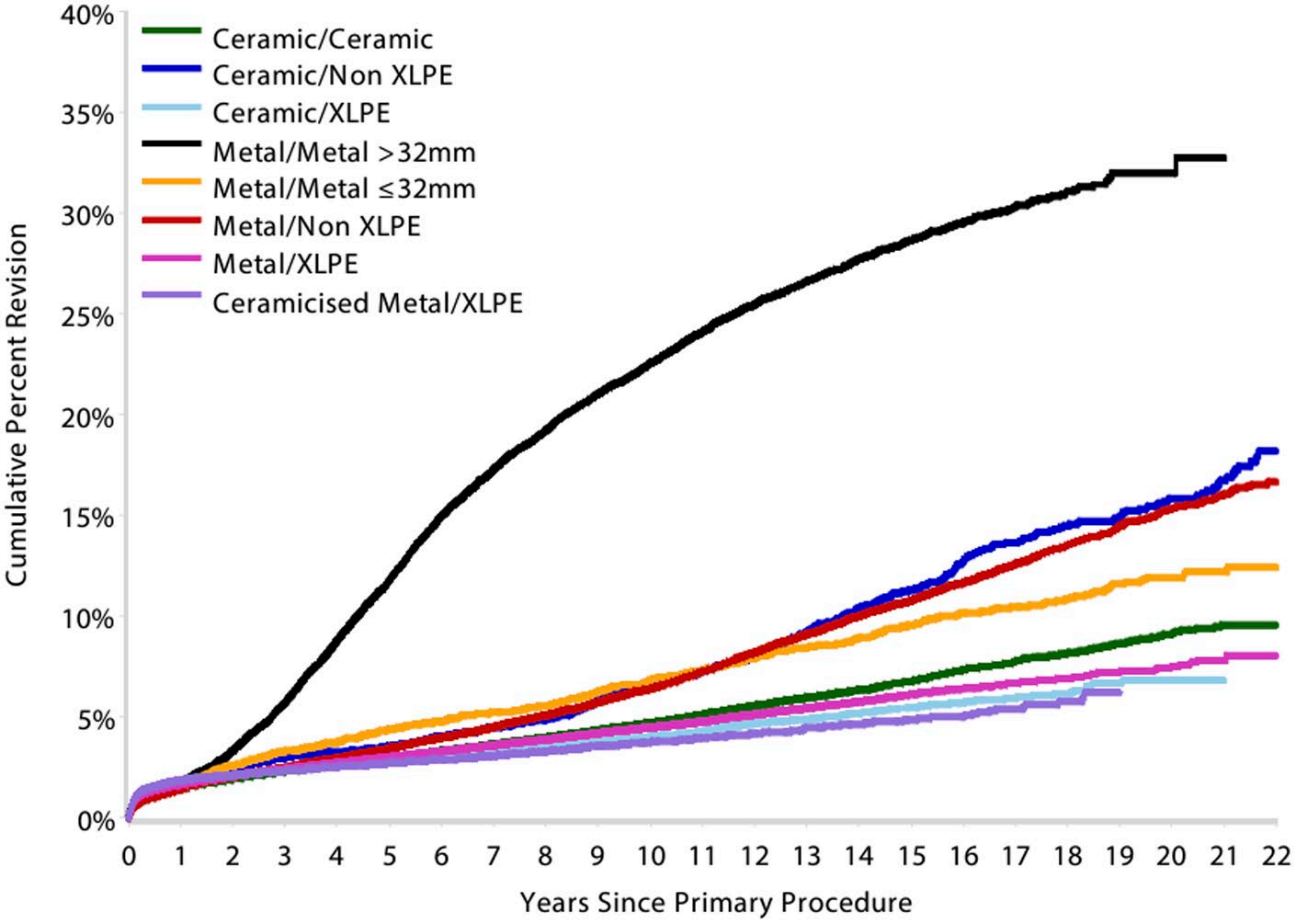
Australian Joint Registry showing comparative wear rates of different bearing surfaces up to 19 years.

The Australian Joint Registry, when comparing different bearing surfaces, has demonstrated the superior survivorship of OxZr (also known as ceramicised metal*) versus XLPE at 19 years (Figure 3).^40^ But the proprietary nature of this means they are cautious with their announcement of this superiority.

*Ceramicised metal/XLPE has the lowest rate of revision at 10 years. However, the results should be interpreted with caution as this bearing is a single company product, used with a small number of femoral stem and acetabular component combinations. This may have a confounding effect, making it unclear if the lower rate of revision is an effect of the bearing surface or reflects the limited combinations of femoral and acetabular prostheses.

The reasons for the improved survivorship of ceramic and ceramicised metal heads is currently being debated. Whether it is due to decreased polyethylene wear because of the surface properties of the ceramic/ceramicised metal heads or whether there is a limitation in the potential for adverse reaction to metal debris (ARMD) or trunnionosis at the head/taper junction is unknown.^41^

To our knowledge, this study is the first to show a significant decrease in wear of XLPE with OxZr heads. This may therefore confirm the reason or one of the reasons for the improved survivorship.

The increased use of both ceramic and ceramicised metal heads against XLPE when compared to traditional metal-XLPE is down to a perceived better survivorship.^34^ However,this comes at an extra cost. Cost-effectiveness has already been quantified for certain ages and at certain costs differentials and may not be appropriate for all ages.^42^

### Limitations of study

The major limitation of this study is the question of whether the difference in wear rates (even if statistically significant) has any clinical significance. However, the reason for better survivorship with either ceramic or OxZr heads has not yet been confirmed and this paper adds to the weight of evidence.

Additionally, this study is also retrospective and may lend itself to bias. The analysis, however, is a validated technique which has already been used many times in this journal.

Additionally, the increased use of both ceramic and ceramicised metal heads against XLPE when compared to traditional metal-XLPE is down to a perceived better survivorship.^34^ But this comes at an extra cost. The cost-effectiveness has already been quantified at certain ages and at certain costs differentials and may not be appropriate for all ages.^42^

When this trial was started, it was not clear what XLPE wear rates would be with larger heads. As a result, the use of smaller heads was still common. There are now multiple publications to show wear rates remain low and constant even with thin polyethylene.^43^ However, this study remains pertinent to reflect overall wear rates.

This is a multicentre study, with at least 5 different surgeons (all performing a posterior approach) which, although it may confer bias, adds to the transferability of the information to the real-world setting. Similarly, patient demographics such as age/a proxy for activity levels and body mass index (BMI) were not included and may have an impact on wear rates. This study only assesses 28-mm heads, the use of which is now on the decline compared to 32- and 36-mm heads. Our assessment methodology is a validated assessment protocol that has been correlated to RSA.

This study is purely a radiological assessment of wear rates and osteolysis risk, and does not reflect function and pain levels.

Finally, the OxZr heads are a proprietary product that is only made by 1 manufacturer (Smith+Nephew).
